# Genomic selection signatures in sheep from the Western Pyrenees

**DOI:** 10.1186/s12711-018-0378-x

**Published:** 2018-03-22

**Authors:** Otsanda Ruiz-Larrañaga, Jorge Langa, Fernando Rendo, Carmen Manzano, Mikel Iriondo, Andone Estonba

**Affiliations:** 1 Genetics, Physical Anthropology and Animal Physiology Department, University of the Basque Country (UPV/EHU), Leioa, Spain; 2 Genetics, Sequencing and Genotyping Unit, Advanced Research Facilities (SGIker), University of the Basque Country (UPV/EHU), Leioa, Spain

## Abstract

**Background:**

The current large spectrum of sheep phenotypic diversity results from the combined product of sheep selection for different production traits such as wool, milk and meat, and its natural adaptation to new environments. In this study, we scanned the genome of 25 Sasi Ardi and 75 Latxa sheep from the Western Pyrenees for three types of regions under selection: (1) regions underlying local adaptation of Sasi Ardi semi-feral sheep, (2) regions related to a long traditional dairy selection pressure in Latxa sheep, and (3) regions experiencing the specific effect of the modern genetic improvement program established for the Latxa breed during the last three decades.

**Results:**

Thirty-two selected candidate regions including 147 annotated genes were detected by using three statistical parameters: pooled heterozygosity H, Tajima’s D, and Wright’s fixation index F_st_. For Sasi Ardi sheep, chromosomes *Ovis aries* (OAR)4, 6, and 22 showed the strongest signals and harbored several candidate genes related to energy metabolism and morphology (*BBS9, ELOVL3* and *LDB1*), immunity (*NFKB2*), and reproduction (*H2AFZ*). The major genomic difference between Sasi Ardi and Latxa sheep was on OAR6, which is known to affect milk production, with highly selected regions around the *ABCG2*, *SPP1*, *LAP3*, *NCAPG*, *LCORL*, and *MEPE* genes in Latxa sheep. The effect of the modern genetic improvement program on Latxa sheep was also evident on OAR15, on which several olfactory genes are located. We also detected several genes involved in reproduction such as *ESR1* and *ZNF366* that were affected by this selection program.

**Conclusions:**

Natural and artificial selection have shaped the genome of both Sasi Ardi and Latxa sheep. Our results suggest that Sasi Ardi traits related to energy metabolism, morphological, reproductive, and immunological features have been under positive selection to adapt this semi-feral sheep to its particular environment. The highly selected Latxa sheep for dairy production showed clear signatures of selection in genomic regions related to milk production. Furthermore, our data indicate that the selection criteria applied in the modern genetic improvement program affect immunity and reproduction traits.

**Electronic supplementary material:**

The online version of this article (10.1186/s12711-018-0378-x) contains supplementary material, which is available to authorized users.

## Background

Sheep is one of the first species that was domesticated approximately 11,000 years ago in the Fertile Crescent [1] due in part to its small size, docile behavior and high adaptability to very different environments. During the following 3000 to 4000 years, sheep spread across Europe, Africa and Asia together with the expansion of the Neolithic culture and the development of agriculture [2]. From the beginning, humans selected sheep for desirable production traits such as wool, milk and meat [3]. This selection process, combined with natural adaptation to new environments, has led to a large spectrum of phenotypic diversity with more than one thousand different sheep breeds currently described [4].

In the Western Pyrenees, two sheep breeds are described: Latxa sheep, also known as Manexa or Manech, and Sasi Ardi. Although these breeds show morphological and genetic similarities [5], there are marked differences in their habitat and breeding systems that make them good candidates for the study of the genetic response to artificial selection and local adaptation.

Most of the Western Pyrenees sheep belong to the Latxa breed, which has good dairy aptitude and is well adapted to the prevailing climatological and orographic conditions in its area of production. Two main varieties of Latxa sheep are distinguished according to facial and extremity pigmentation [6]: Latxa Blonde Face and Latxa Black Face. Latxa sheep have been traditionally selected for increased milk production; with the introduction of modern quantitative genetics methodologies, a genetic improvement program for this breed was established in 1981. Initially, the selection goal was to increase milk production in order to improve the profitability of the flocks. Later, milk composition and udder morphology traits were also included among the major selection criteria of the program, together with resistance to transmissible spongiform encephalopathies.

In contrast to Latxa sheep, Sasi Ardi is a scarcely known semi-feral breed, which is highly adapted to wooded mountainous areas that are poorly accessible to humans or to other sheep breeds [7]. Because of this specificity, Sasi Ardi sheep represent an environmentally and socially important breed by contributing largely to the clearing and cleaning of the understory, thus preserving the environment from fires and maintaining the landscape. Sasi Ardi sheep have a smaller body size than Latxa, with slightly elongated extremities, a uniform blonde or reddish color, and a straight wool-less neck. Although there is no specific breeding scheme for production traits in Sasi Ardi sheep, currently it is mainly bred for meat production. These sheep are raised in an extensive production system with grazing as the only food source [7], and thus, natural selection is the main evolutionary factor that has driven the genetic pool of the breed. Since 1997, Sasi Ardi is considered as an autochthonous breed under special protection, and since 2007 as a breed in danger of extinction (Official Catalog of Spanish Livestock Breeds; http://www.mapama.gob.es/es/ganaderia/temas/zootecnia/) [8]. This situation has slightly improved during the last years thanks to the implementation in 2007 of the Conservation Program for Sasi Ardi breed. This program aims at preserving the breed while maintaining its genetic variability by avoiding consanguineous mating.

Based on the above considerations, the main objective of our study was to assess the impact of artificial and natural selection on the genetic pool of the two Western Pyrenees sheep breeds, through genome-wide selection scans (GWSS) to: (1) understand the genetic basis of the local adaptation of Sasi Ardi semi-feral sheep, (2) decipher the genomic response in Latxa sheep caused by a long traditional selection pressure, and (3) explore the specific genomic effect of the genetic improvement program implemented in the Latxa breed.

## Methods

### Whole-genome resequencing

Seventy-five female DNA samples from three populations were sequenced following the Pool-Seq approach [9]: 25 Sasi Ardi (SAS), 25 Latxa individuals included in the genetic improvement program (LAS), and 25 Latxa individuals not included in the genetic improvement program (LAN). All analyzed Latxa individuals belonged to the Latxa Blonde Face ecotype. DNA was extracted with the NucleoSpin^®^ 96 Blood Core Kit (Macherey–Nagel) commercial kit and pooled in equimolar quantities (4 µl at 20 ng/µl). Three DNA libraries were generated, one for each pooled population, using the TruSeq DNA PCR-Free Sample Preparation Kit of Illumina (fragment size average 350 bp) and sequenced on a HiSeq 2000 instrument (paired-end, 2 × 100 nt) according to the manufacturer’s instructions.

### Data processing

Illumina adaptors and low-quality bases were removed by applying the Trimmomatic v0.32 program [10] with the following parameters: AVGQUAL:3 ILLUMINACLIP:TruSeq 3-PE-2.fa:2:30:10 MINLEN:31 LEADING:19 TRAILING:19 MINLEN:31. High-quality sequences were mapped against the domestic sheep reference assembly *Ovis aries* (OAR) v3.1 [11] with Bowtie2 v2.2.3 [12], using the –no-unal option. SAMtools v0.1.19 [13] and Picard-tools v1.124 (http://broadinstitute.github.io/picard/) were used to convert between SAM and BAM formats (samtools view), remove duplicate reads (picard-tools MarkDuplicates), sort the BAM file (picard-tools SortSam), remove reads with low-quality mapping (MAPQ quality score < 20 and retain only pairs that are properly mapped: samtools view − q 20 − f 2 − F 4 − F 8), and to perform proper variant calling (samtools mpileup -B -Q 0). Finally, indels were also removed using the filter-pileup-by-gtf.pl Perl script from the PoPoolation package [14]. Each step in this data processing was carried out separately for each population.

### Genome-wide selection scan (GWSS)

For the identification of selective sweep regions, i.e. genomic regions with patterns that are consistent with positive selection, both within- and between-population analyses were performed for the three sequenced populations. A sliding window approach was carried out, in which a single nucleotide polymorphism (SNP) was called if at least two reads were present for the non-reference allele. An appropriate window size of 200 kb (with sliding steps of 50 kb) was previously determined in an in silico simulation study [see Additional file 1] in order to avoid a maximum number of windows with less than 10 SNPs that may lead to a possible bias in the estimation of parameters used for the detection of selection signatures [15, 16].

#### Within-population analysis

Two statistical parameters were calculated for each population: pooled heterozygosity (H_p_) and pooled Tajima’s D (D_p_). This approach has already been applied in selection mapping studies for various domestic species including sheep [15–18]. Pooled heterozygosity (H_p_) was calculated by running a Python3 script and using the formula H_p_ = 2Σn_MAJ_ Σn_MIN_/(Σn_MAJ_ + Σn_MIN_)^2^ [17], where Σn_MAJ_ is the sum of the major allele reads, and Σn_MIN_ the sum of the minor allele reads for all SNPs in each window.

In addition, the pooled Tajima’s D (D_p_) statistics [19] was calculated for the three sequenced populations (SAS, LAS, and LAN) to explore the possible distortions in the distribution of allele frequencies using the ‘corrected’ method implemented in the PoPoolation software [14]. Tajima’s D parameter compares the difference between the mean pairwise difference and the number of segregating sites to detect selection signatures [19].

#### Between-population analysis

In order to detect strong recent selection signatures, we compared the genome of the three populations searching for regions with increased genetic distance between them, by estimating Wright’s fixation index F_st_ [20]. Thus, pooled F_st_ values i.e. F_stp_ were estimated for SAS versus LAS, SAS versus LAN, and LAS versus LAN comparisons using the Perl scripts from PoPoolation [21].

### Identification of selected candidate regions (SCR)

Autosomal H_p_, D_p_, and F_stp_ distributions were Z-transformed, resulting in Z(H_p_), Z(D_p_), Z(F_stp_). Windows were defined as significant if they were at least six standard deviations away from the mean Z scores: i.e. Z(H_p_) and Z(D_p_) lower than − 6 for the within-population analysis, and/or Z(F_stp_) higher than 6 for the between-population analysis, since these values represent the extreme lower and upper tails of the distributions. Then, consecutive significant windows were considered as representative of putative selective sweeps and named selected candidate regions (SCR). In order to avoid excess fragmentation of predicted selective sweeps, regions that were separated by one or two windows that did not meet the above extension criteria were collapsed into a single putative sweep region.

SCR were classified into three groups: (A) SCR specific to Sasi Ardi (within-population analysis of SAS), (B) SCR common to the two analyzed Latxa populations (within-population analysis of LAS and LAN), and (C) SCR assumed to result from the intensive artificial selection applied during the Latxa sheep improvement program (between-population analysis LAS *vs*. LAN).

### Gene annotation and enrichment analysis within SCR

SCR were extended 100 kb up- and downstream in order to include potential effects of regulatory changes on loci and to reduce the risk of excluding the outermost portions of the selected haplotypes by using sliding windows of fixed size [16]. The *Ovis aries* 3.1 genome assembly from the Ensembl’s Biomart website (http://www.ensembl.org/biomart) [22] was used for the identification of annotated genes in SCR. The sheep QTL database [23] available online at http://www.animalgenome.org/cgi-bin/QTLdb/BT/search was searched to identify SCR that overlapped with previously published sheep QTL.

GO terms, including biological process, cellular component, and molecular function, and KEGG pathway enrichment analyses were performed on the three SCR groups, using the function annotation clustering tool from the Database for Annotation, Visualization and Integrated Discovery (DAVID v6.8, http://david.abcc.ncifcrf.gov/) [24, 25]. Because the annotation of the ovine genome is still incomplete, orthologous human gene ID for each gene within the detected SCR were analyzed and enrichment analysis of these genes was performed using the human genome background supplied by the DAVID database. Corrections for multiple testing were performed by applying the Benjamini–Hochberg method [26], and GO terms and the KEGG pathways were considered significant at a *P* value lower than 0.05. In the case of functional clusters, an enrichment score of 1.3 (equivalent to Fisher exact test *P* value of 0.05) was used as a threshold, as recommended by the authors of the database.

## Results

A total of 46 Gbp, 42.6 Gbp, and 43.4 Gbp were sequenced for SAS, LAS, and LAN populations, respectively, which resulted in an average read depth of ∼ 16× per pool after trimming. On average, 95.8% of the sequence reads aligned to the Oar_v3.1 reference genome.

**Fig. 1:**
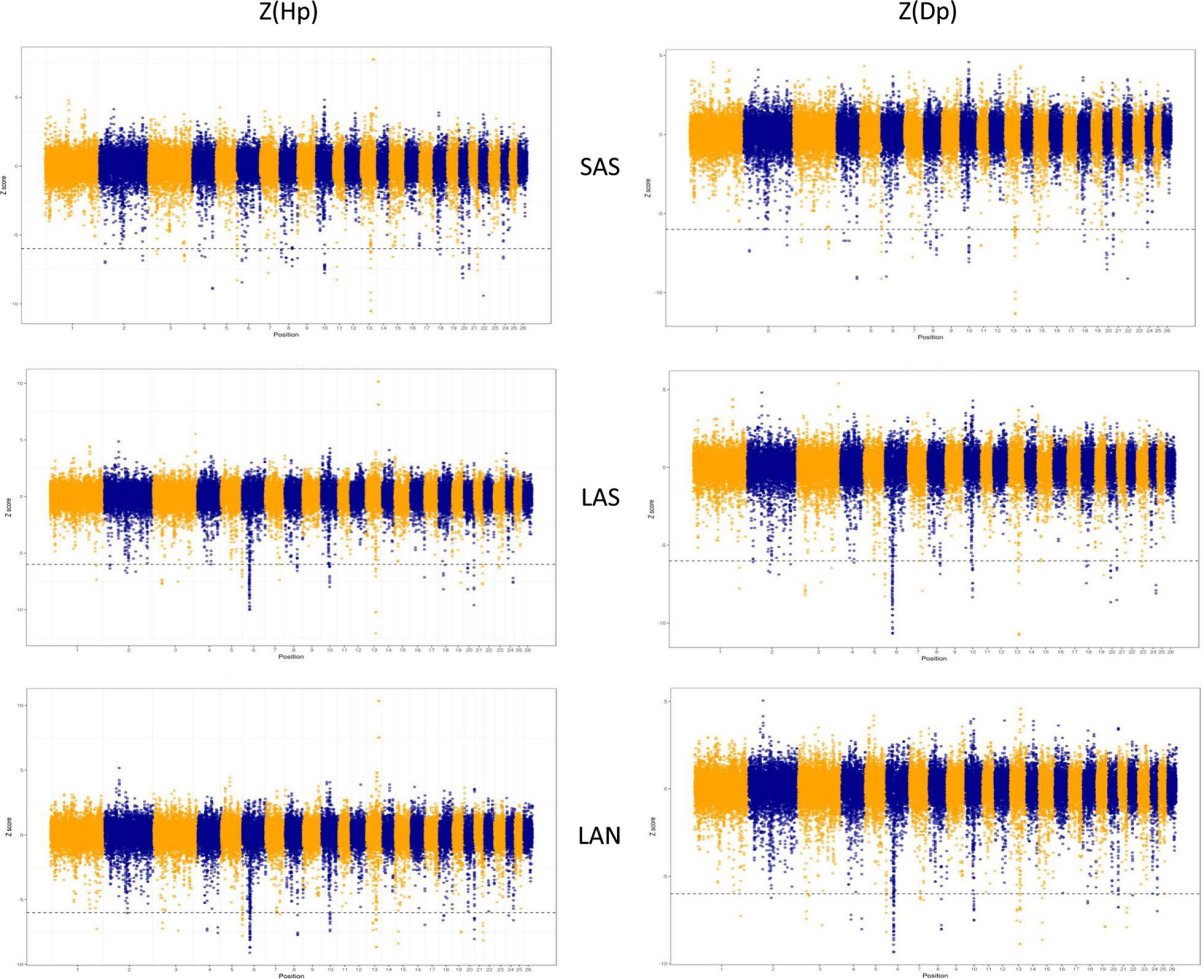
Distribution of Z-transformed average pooled heterozygosity (Z(H_p_)) and pooled Tajima’s D (Z(D_p_)) plotted along sheep autosomes 1 to 26 (alternately colored) for Sasi Ardi (SAS) and both Latxa populations either included in the genetic improvement program (LAS) or not (LAN). A dashed horizontal line indicates the cut-off (Z = − 6) used for extracting outliers

**Fig. 2:**
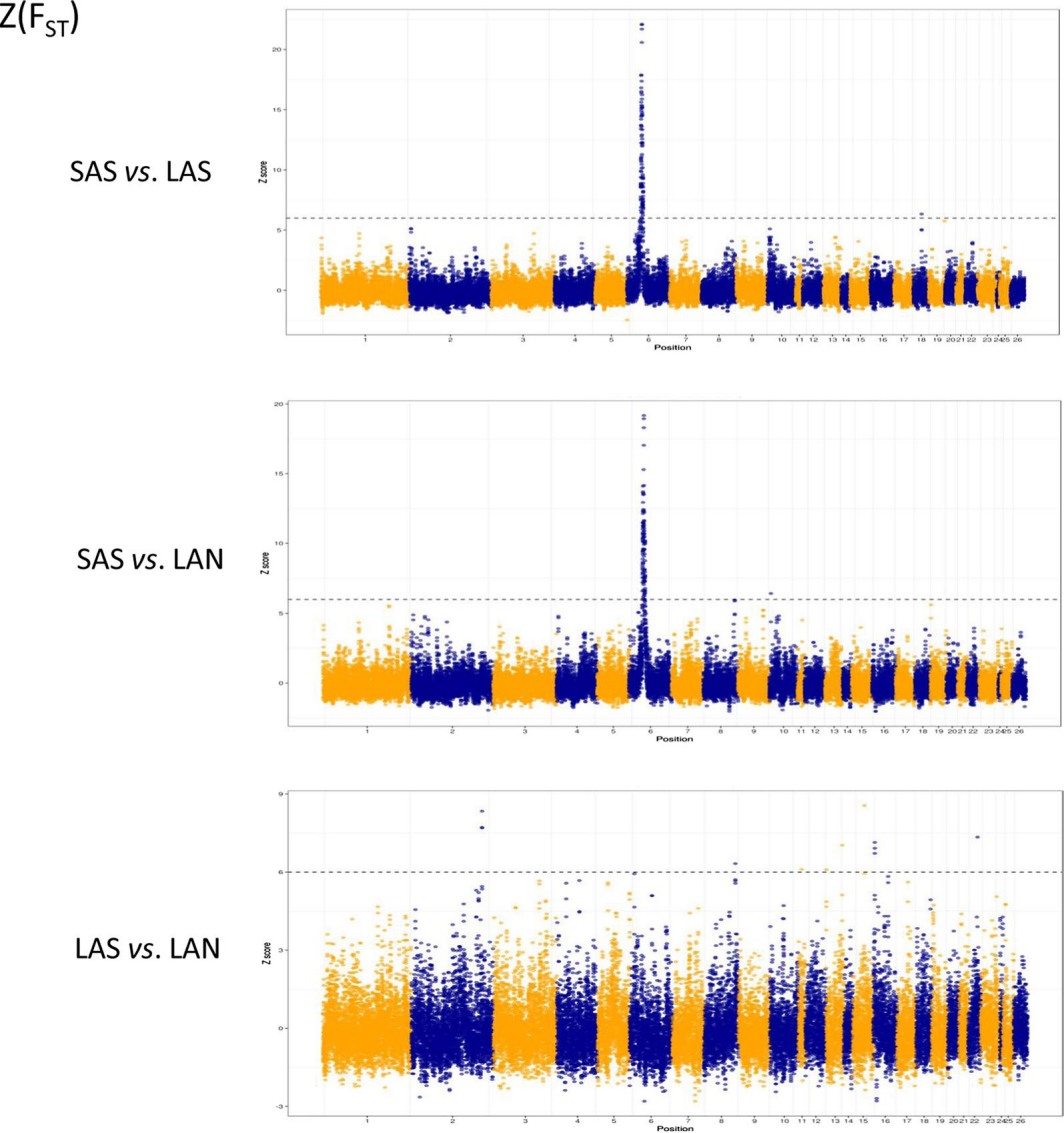
Distribution of Z-transformed average pooled fixation index (Z(F_STp_)) plotted along sheep autosomes 1 to 26 (alternately colored) for Sasi Ardi (SAS) and both Latxa populations either included in the genetic improvement program (LAS) or not (LAN). A dashed horizontal line indicates the cut-off (Z = 6) used for extracting outliers

For the GWSS analysis and the within-population analysis, 11,135,221, 10,384,557, and 10,505,944 SNPs along the 26 ovine autosomes for the SAS, LAS and LAN populations, respectively, were considered to calculate parameters H_p_ and D_p_. In the case of the between-population analysis, 12,366,133 common SNPs in the three populations were included for the estimation of F_stp_. Figures 1 and 2 show plots of the Z(H_p_), Z(D_p_), and Z(F_STp_) test results. Based on the calculation of Z(H_p_) and Z(D_p_) parameters, 82, 108, and 85 significant windows were identified for SAS, LAS, and LAN populations, respectively, which corresponded to 0.16, 0.22, and 0.17% of the test results [see Additional file 2]. The genetic differentiation analysis (SAS versus LAS, SAS versus LAN, and LAS versus LAN) revealed 99, 106, and 12 significant windows (Z(F_stp_) > 6) corresponding to 0.31, 0.33, and 0.037% of the test results, respectively [see Additional file 3].

Thirty-two SCR were identified within the identified significant windows. The average length of the detected SCR was 385 kb (ranging from 50 kb to 1.95 Mb). The detected SCR were classified into three groups: (A) 16 SCR that were specific to Sasi Ardi sheep (SCR 1 to 16) and located on *Ovis aries* (OAR) chromosomes OAR2, 3, 4, 6, 7, 8, 11, 19, 20, 22, and 24; (B) eight SCR that were common to the two Latxa populations (LAS and LAN) (SCR 17 to 24) and located on OAR1, 2, 3, 6, 7, 18, 19, and 24; and (C) eight SCR (SCR 25 to 32) on OAR2, 8, 11, 13, 15, 16, and 22, which corresponded to the genomic differences between LAS and LAN and are thought to be genomic regions that are affected by the intensive artificial selection applied in the Latxa sheep genetic improvement program (Table 1).

**Table 1:** Identified SCR classified in three groups: (A) specific to Sasi Ardi (SAS) sheep based on the outliers for Z(H_p_) and Z(D_p_) distribution, (B) common to the two Latxa populations (LAS and LAN) based on the outliers for Z(H_p_) and Z(D_p_) distribution, and (C) based on the outliers for Z(Fst_p_) distribution when comparing both Latxa sheep populations (LAS vs. LAN)

Group	SCR	Chr	Start (kb)	End (kb)	Number of genes	Candidate genes and selected traits^a^
A	SCR1	2	219,400	219,650	14	*SLC11A1* (immunity)
	SCR2	3	182,650	183,050	5	
	SCR3	4	35,150	35,350	2	
	SCR4	4	63,050	63,250	3	*BBS9* (metabolism)
	SCR5	6	24,650	24,950	7	*H2AFZ* (reproduction)
	SCR6	6	44,750	45,000	7	
	SCR7	6	48,300	48,500	3	
	SCR8	7	42,000	42,250	5	*NID2* (reproduction)
	SCR9	8	7950	8300	0	
	SCR10	8	31,900	32,250	4	
	SCR11	8	90,100	90,300	6	
	SCR12	11	18,300	18,550	5	
	SCR13	19	29,600	29,800	1	
	SCR14	20	15,050	15,300	11	*TREM1* and *TREM2* (immunity)
	SCR15	22	22,050	22,250	15	*NFKB2* (immunity), *LDB1* (metabolism), *ELOVL3* (metabolism)
	SCR16	24	10,250	10,500	10	
B	SCR17	1	234,800	23,5000	8	*GPR171* (feeding and metabolism)
	SCR18	2	115,000	115,200	0	
	SCR19	3	124,800	125,000	1	*KITLG* (pigmentation/reproduction/milk production)
	SCR20^b^	6	36,450	38,400	19	*ABCG2, SPP1, LAP3, NCAPG,* and *MEPE* (milk production), *NCAPG* and *LCORL* (body size)
	SCR21	7	72,550	72,600	7	
	SCR22	18	23,600	23,850	3	
	SCR23	19	43,300	43,500	5	
	SCR24	24	34,650	34,800	6	*SH2B2* (body size)
C	SCR25	2	202,050	202,350	12	*BZW1* (reproduction)
	SCR26	8	75,350	75,550	1	*ESR1* (reproduction)
	SCR27	11	18,350	18,550	5	
	SCR28	13	350	550	0	
	SCR29	13	58,750	58,950	14	
	SCR30	15	46,150	46,350	20	*OR56A1, OR52W1, OR52B2, OR56B4, OR56A3, CNGA4* (scrapie)
	SCR31	16	9050	9350	6	*ZNF366* (reproduction)
	SCR32	22	29,050	29,250	2	

*SCR* selected candidate regions; *chr* chromosome number

^a^SCR were extended 100 Kb up- and downstream for gene annotation

^b^Three continuous SCR regions on OAR6 were merged: 36,450,000 bp to 37,000,000 bp, 37,050,000 bp to 38,100,000 bp, and 38,200,000 bp to 38,400,000 bp

The detected SCR included 147 annotated genes (on average six genes per SCR) (Table 1). The DAVID functional analysis did not yield any significantly enriched gene ontology or KEGG pathway term for the 16 SCR that were specific to Sasi Ardi sheep. Nevertheless, the genes within the SCR that were common to the two analyzed Latxa populations showed two significantly enriched GO terms (Benjamini–Hochberg *P* < 0.05, Table 2): GO00045028 (G-protein coupled purinergic nucleotide receptor activity) and GO0035589 (G-protein coupled purinergic nucleotide receptor signaling pathway). Both categories included five genes within SCR17 located on OAR1: *P2RY12*, *P2RY13*, *P2RY14*, *GPR171*, and *GPR87*. These terms, together with GO0005887 and GO0007186, form a significantly enriched functional cluster (enrichment score = 4.61) related to signal transduction. For the SCR of group C, which may result from the improvement program applied in Latxa sheep, we identified two significantly enriched categories: hsa04740 KEGG pathway (olfactory transduction) and GO0004984 (olfactory receptor activity). All genes involved in these two terms were within SCR30 located on OAR15: *OR56A3*, *OR56A4*, *OR52B2*, *OR56A1*, *OR56B4*, *OR52L1*, *OR52W1*, and *CNGA4*. The two categories were included in a significantly enriched functional cluster (enrichment score = 2.72) in which other terms related to the detection of chemical stimulus and sensory perception, signal transduction, and receptor activity are found.

**Table 2:** Significantly enriched annotation clusters and functional terms

Annotation cluster 1	Enrichment score: 4.6		
Category	Term	Genes	Corrected *P* value
*SCR group B*			
GOTERM_MF_DIRECT	GO:0045028: G-protein coupled purinergic nucleotide receptor activity	*P2RY12*, *P2RY13*, *P2RY14*, *GPR171*, *GPR87*	3.7 × 10^−7^
GOTERM_BP_DIRECT	GO:0035589: G-protein coupled purinergic nucleotide receptor signaling pathway	*P2RY12*, *P2RY13*, *P2RY14*, *GPR171*, *GPR87*	1.3 × 10^−6^
GOTERM_CC_DIRECT	GO:0005887: integral component of plasma membrane	*P2RY12*, *P2RY13*, *P2RY14*, *SLMAP*, *GPR171*, *PKD2*, *GPR87*	0.790
GOTERM_BP_DIRECT	GO:0007186: G-protein coupled receptor signaling pathway	*P2RY12*, *P2RY14*, *GPR171*	1.000

Annotation cluster 1	Enrichment score: 2.7		
Category	Term	Genes	Corrected *P* value
*SCR group C*			
KEGG_PATHWAY	hsa04740: olfactory transduction	*OR56A3*, *OR56A4*, *OR52B2*, *OR56A1*, *OR56B4*, *OR52L1*, *OR52W1*, *CNGA4*	0.002
GOTERM_MF_DIRECT	GO:0004984: olfactory receptor activity	*OR56A3*, *OR56A4*, *OR52B2*, *OR56A1*, *OR56B4*, *OR52L1*, *OR52W1*	0.027
GOTERM_BP_DIRECT	GO:0050911: detection of chemical stimulus involved in sensory perception of smell	*OR56A3*, *OR56A4*, *OR52B2*, *OR56A1*, *OR56B4*, *OR52L1*, *OR52W1*	0.101
GOTERM_BP_DIRECT	GO:0007165: signal transduction	*OR56A3*, *OR52B2*, *OR56B4*, *OR52L1*, *EVI2A*, *NF1*, *SMPD1*, *OR52W1*, *ESR1*, *APBB1*	0.130
GOTERM_MF_DIRECT	GO:0004930: G-protein coupled receptor activity	*OR56A3*, *OR56A4*, *OR52B2*, *OR56A1*, *OR56B4*, *OR52L1*, *OR52W1*	0.163
GOTERM_BP_DIRECT	GO:0007186: G-protein coupled receptor signaling pathway	*OR56A3*, *OR52B2*, *OR56B4*, *OR52L1*, *CCKBR*, *OR52W1*	0.980
GOTERM_CC_DIRECT	GO:0005886: plasma membrane	*OR52B2*, *CCKBR*, *MAP1B*, *NSFL1C*, *ESR1*, *OR56A3*, *OR56A4*, *OR56B4*, *OR56A1*, *OR52L1*, *SMPD1*, *OR52W1*, *MC3R*, *OMG*, *APBB1*, *FAM126B*	0.964

## Discussion

With the advance of high-throughput genotyping and sequencing technologies, the analysis of large datasets offers great opportunities to study genome evolution in response to selection forces in both wild and domesticated species. In recent years, selection mapping studies have become increasingly popular because they offer a complementary strategy to genome-wide association studies (GWAS) for mapping variants that impact traits of interest, and thus help to link phenotypes to gene function, which could be of biotechnological relevance.

Our study constitutes the first genome-wide comparison of Sasi Ardi and Latxa local sheep from the Western Pyrenees. First, we resequenced the genomes of these two breeds by using Illumina’s technology following a Pool-Seq approach [9]. Then, we calculated different within- and between-population parameters with the aim of detecting selective sweeps in the genome and 32 SCR were identified.

### Natural selection and Sasi Ardi genome

Understanding the molecular basis of local adaptation is one of the major challenges facing population genetics. With this in mind, our first objective was to explore the genome of Sasi Ardi sheep (SAS) to search for selection signatures that underlie natural adaptation of this breed to the specific environment in which it lives in the Western Pyrenees. We detected 16 SCR specific to this breed, among which the strongest signals were detected on OAR4 (SCR3 and 4), OAR6 (SCR5), and OAR22 (SCR15). The functions of some candidate genes identified within these regions indicate that natural selection may have affected energy metabolism and morphology, immunity, and reproduction traits of Sasi Ardi semi-feral sheep.

A recent worldwide study suggested that climate exerts a selective pressure on genes related to energy metabolism in sheep [27]. Sasi Ardi and Latxa sheep are both autochthonous breeds of the Western Pyrenees, but the semi-feral nature of the Sasi Ardi breed and its harsh habitat, suggest that these conditions are more demanding than those of the Latxa, as might be reflected in its smaller body and thin elongated limbs. We found several candidate genes (*BBS9, ELOVL3* and *LDB1*) that support this hypothesis. The *BBS9* (*Bardet*-*Biedl syndrome 9*) gene located within SCR4 constitutes one of the 15 loci that are associated with Bardet-Biedl syndrome (BBS), which in humans is a genetically heterogeneous disorder characterized by marked obesity among other clinical features [28]. Mutations in several *BBS* genes affect fat cell differentiation, and recently this gene has been specifically associated to abdominal visceral fat depots in humans [29]. The *ELOVL3* (*elongation of very long chain fatty acids 3*) gene within SCR15 belongs to a family of genes that play a role in the regulation of energy metabolism [30]. Specifically, *ELOVL3* is expressed in brown adipose tissue and skin, and in mouse, mutations in this gene lead to several abnormalities in sebaceous lipid composition, impaired skin defects and metabolic irregularities in adipose tissue [31]. Finally, the protein encoded by the *LDB1* gene (*LIM*-*domain binding coregulator*), also located within SCR15 binds to ELOVL3 and participates in energy homeostasis in mice [32].

It is well known that immunity and host response genes and functions are recurrent targets of natural selection, both in humans [33] and free-living animal species [34]. In our study, the results from the Sasi Ardi genome analysis reveal several interesting genes related to the immune system. The putatively selected SCR15 contains the gene that encodes the transcription factor NFKB2 (nuclear factor of kappa light polypeptide gene enhancer in B-cells 2), which is involved primarily in immunity, among other processes [35]. In previous studies, several quantitative trait loci (QTL) have been detected within SCR15 and are related to animal health, among which some are associated with response to parasitic infections, although no candidate gene has yet been suggested [36, 37]. In addition, selection signatures within SCR1 and SCR14, although not as significant as that within SCR15, also harbor candidate genes relevant to immunity. These include genes that are involved in triggering receptors expressed on myeloid cells (TREM), *TREM1* and *TREM2* in the present study, which interact with microbial products that activate innate immunity responses of organisms [38]; and the *SLC11A1* gene (*solute carrier family 11 member 1*), which encodes an iron transporter and also plays a key role in innate immunity against infection by intracellular pathogens [39]. *SLC11A1* has been extensively associated with natural resistance to several infections in ruminants, including the *Mycobacterium avium* subsp. *paratuberculosis* infection in both cattle and sheep [40, 41]. Interestingly, a recent study in *Equidae* identified several codons in the *SLC11A1* gene under positive selection [42].

Another candidate gene under putative selection is *H2AFZ* (*histone H2A.Z*), located within SCR5. *H2AFZ* is required for early mammalian development [43], and in pig, it is related to litter size [44, 45]. Sasi Ardi ewes are characterized by low fertility and small litter size, due, in part, to the harsh environment in which the breed is raised [7]. Thus, the idea of natural selection affecting the *H2AFZ* gene and shaping reproduction traits of Sasi Ardi sheep seems plausible. The presence of the *NID2* (*nidogen 2*) gene within SCR8 further supports this hypothesis, since modulation of *NID2* in the endometrium has been suggested to have a role in establishing uterine receptivity to implantation in cattle [46].

### Effects of selection for milk production in Latxa sheep

After the early stages of domestication of livestock species, methodical selection of specific traits was undertaken [3]. Unlike the Sasi Ardi breed, traditionally, Latxa sheep are exploited for milk production, and selection for this trait was much intensified during the last three decades through a genetic improvement program. The consequences of this selection were clearly reflected in the Z(H_p_) and Z(D_p_) values obtained from the within-population analysis of both LAS and LAN (Fig. 1) in which OAR6, a well-known milk related chromosome, showed highly significant values. In fact, selection signatures on OAR6 constituted the main genetic difference when comparing SAS with both LAS and LAN (Fig. 2). *Bos taurus* (BTA) chromosome BTA6 is well known for its association with milk production in dairy cattle [47]. In sheep, several QTL for milk production traits have also been described on the orthologous chromosome OAR6, which overlap with the SCR20 detected in the current study [48]. Several studies have identified this genomic region as being under selection in dairy breeds [3, 4, 18, 49–51]. SCR20 includes several genes that are considered to be putatively selected for milk production traits in sheep. *ABCG2* (*ATP*-*binding cassette, sub*-*family G, member 2*) is a well-known gene that has a role in milk yield and composition and is under selection in both dairy cattle [52–54] and sheep [3, 4, 18]. The *SPP1* (*secreted phosphoprotein 1* or *osteopontin*) gene is assumed to regulate lactation in cattle [55] and is suggested to be putatively under selection in sheep [18, 49]. Furthermore, within this region, *LAP3* (*leucine aminopeptidase 3*) is associated with milk production traits in cattle [56, 57], *NCAPG* (*non*-*SMC condensin I complex, subunit G*) is reported to influence milk fat yield [47], and *MEPE* (*matrix extracellular phosphoglycoprotein*) is suggested to be a candidate gene for fat and protein percentage [58].

Interestingly, the genes associated with SCR20 point to another hypothesis regarding the putative selection of this genomic region. At the beginning of the domestication process, the body size of animals decreased substantially, but as artificial selection for production was applied it increased again considerably [50]. Related to this, the previously mentioned *NCAPG* gene and the *LCORL* (*ligand dependent nuclear receptor corepressor like*) gene encode transcriptional regulators that affect body size traits in Merino sheep [59]. Both genes *NCAPG* and *LCORL*, have also been described as putatively under selection when comparing dairy, including Latxa, and non-dairy Spanish sheep breeds [50], although the authors could not explain this finding due to the lack of differences in body size traits between the breeds analyzed. However, in our study, the hypothesis of selection acting on body size is supported by the presence of the *SH2B adapter protein 2* (*SH2B2*) gene within SCR24. Although there is little evidence in the literature, Yang et al. [60] reported an association between the bovine ortholog *SH2B2* and growth performance in Nanyang cattle, which suggests a possible role through the regulation of glucose uptake.

The identification of SCR19 in Latxa sheep is also of interest. The *KIT ligand gene* (*KITLG*), located 40 kb upstream from this SCR, encodes a key controlling receptor for a number of cell types, including hematopoietic stem cells, mast cells, melanocytes and germ cells [61]. This functional complexity offers different hypotheses in the present context. On the one hand, the *KITLG* gene has been associated with coat color in domestic animals [62–64], including sheep in which two candidate regions around the *KIT* and *KITLG* genes were identified [3, 4]. In our study, the putative selection of *KITLG* in Latxa sheep could be responsible for the differences in facial and extremity pigmentation between its two main ecotypes, i.e. the Latxa Black Face and the Latxa Blonde Face [6]. On the other hand, the putative selection signature observed near *KITLG* could be related to its fundamental impact on reproduction traits. In fact, pleiotropic effects of *KIT* and *KITLG* on the reproductive tract have been described in different species [65]. The antagonistic genetic correlation between female fertility and milk production is well documented in cattle and sheep [66–68] and is supported by a study in Latxa sheep [69] that reported that, in this breed strongly selected for dairy production, young ewes had low average fertility.

The SCR17, which was detected in both Latxa sheep populations, contains several genes that are related to signal transduction and belong to the two significantly enriched GO categories detected in this study. These genes (*P2RY12*, *P2RY13*, *P2RY14, GPR171,* and *GPR87*) belong to the family of *G protein*-*coupled receptors*. This type of receptor is activated by a wide spectrum of extracellular stimuli, including photons, ions, neurotransmitters, lipids, chemokines and hormones, and binds to G proteins to initiate downstream signaling networks, resulting in a broad range of physiological and pathological processes [70]. In particular, the neuropeptide receptor GPR171 plays an important role in responses associated with feeding and metabolism in mice [71]. This finding might also be relevant in the case of Latxa sheep for which a correlation of feeding and metabolism traits with milk composition was reported [72].

### Consequences of more than 30 years of genetic improvement in Latxa

Thus far, SCR common to both Latxa populations in our study have been interpreted as being the result of traditional selection for milk production. Hereafter, we consider the specific effect of the intensive artificial selection that has been applied since 1981 through the established genetic improvement program. By comparing both Latxa populations, we identified several genomic regions under putative selection (LAS vs. LAN, SCR 25 to 32). Among these, SCR30 showed the most significant signal. This genomic region contains several genes related to the olfactory system including a number of olfactory receptor genes (*OR56A1, OR52W1*, *OR52B2*, *OR56B4*, *OR56A3, OR56A4,* and *OR52L1*), and the *CNGA4* (*cyclic nucleotide gated channel alpha 4*) gene which encodes a subunit of the olfactory CNG channels that are involved in the signal transduction of olfactory sensory neurons in vertebrates [73]. These genes within SCR30 constitute the most significantly enriched category. Interestingly, Vitezica et al. [74] showed that genes involved in the olfactory system are associated with scrapie in sheep, and Ugarte [75] reported that, since 2003, specific genotypes for the *PRNP* (*prion protein*) gene are under selection in the program to increase resistance to scrapie. Our findings suggest selection for genetic resistance to scrapie has an indirect effect on the allelic frequencies of other putative scrapie-related genes such as olfactory receptor genes.

Our results also suggest that reproduction traits may be affected by the genetic improvement program in Latxa sheep. On the one hand, the detrimental consequences on reproduction traits of the long traditional selection for milk production discussed above may have been intensified with the implementation of this program. On the other hand, the reproductive activity of the animals under selection has been altered by the production system, which tends to stimulate reproduction outside of the natural breeding season due to hormonal treatments [69]. Naturally, selection signatures within regions that contain reproduction-related genes could also be explained by the objective of any breeding scheme to produce healthy and fertile individuals that are capable of nurturing their offspring [76]. In our study, we found several reproduction candidate genes in various SCR, including two estrogen receptors genes: *ESR1* (*estrogen receptor 1*) and *ZNF366* (*zinc finger protein 366*). In addition, the transcriptional regulator *BZW1* (*basic leucine zipper and W2 domains 1*) gene located within SCR25 has been linked to bovine endometriosis [77].

### Methodological considerations

In this study, we found little overlap between within-population (H_p_ and D_p_) and between-population (F_stp_) parameters. Only OAR6 harbored a large selected candidate region in both Latxa sheep populations, which constituted the major genomic difference with Sasi Ardi sheep. The absence of overlapping results between the different tests may be explained by their sensitivity to the different selection pressures [78–80]. Furthermore, a recent study that compared the performance of 11 different procedures for detecting selection signatures including Tajima’s D and F_st_ reported low correlations [79]. This is not surprising if we consider that selection signatures based on a reduction of genetic diversity are known to persist for long periods and, therefore, methods such as Tajima’s D or heterozygosity (H) can inform us about old selection events, while the F_st_ statistic, focused on the differentiation between populations, identifies more recent selection processes [79]. Indeed, artificial selection for milk production implemented in the Latxa sheep, both historically and more recently, has led to a selection signal on OAR6 that was detected by both within- and between-population analyses. Moreover, estimated H_p_ and D_p_ parameters identified several genomic signals suggestive of ancient selection events, such as those putatively associated to the local adaptation of Sasi Ardi sheep. The genetic differences observed between both Latxa sheep populations based on F_st_ presumably highlight the effects of the current intensive artificial selection program.

## Conclusions

With the aim to understand the genotype–phenotype relationship better, this study evaluates the impact of selective processes on the sheep genome. A genome-wide comparison of two local sheep breeds from the Western Pyrenees: the semi-feral breed Sasi Ardi, and its highly selected relative Latxa breed, is presented. Genome-wide selection scans highlighted multiple candidate regions in both breeds that contain genes that are relevant to the traits under selection. These findings demonstrate the appropriateness of this approach to achieve our objectives. In Sasi Ardi sheep, natural selection appears to have favored various specific traits related to energy metabolism, morphology, reproduction, and the immune system, making this semi-feral breed suitable for the harsh environment in which it lives. The traditional artificial selection exerted on the Latxa sheep for milk production was clearly detected around well-known genomic regions. In addition, immunity and reproduction traits, might also be affected by the current genetic improvement program.

Our findings provide insight into genes under putative selection in sheep that are subject to different management regimen. Nevertheless, further investigation is required to validate these findings, which should apply complementary methods, such as gene expression analyses, to understand the selection pressures better.

## Additional files

**Additional file 1.** Details of the in silico simulation study that was carried out to determine the appropriate window size to be used for GWSS analysis.

**Additional file 2.** Raw data for Z(H_p_) and Z(D_p_): raw data for pooled heterozygosity (Z(H_p_)) and pooled Tajima’s D (Z(D_p_)) for each analyzed window in each sequenced pool (SAS, LAS, and LAN).

**Additional file 3.** Raw data for Z(F_STp_): raw data for pooled fixation index (Z(F_STp_)) for each analyzed window in each comparison (SAS *vs*. LAS, SAS *vs*. LAN, and LAS *vs*. LAN).
