# Supplementary material for: Genomic selection signatures in sheep from the Western Pyrenees
Source: Genet Sel Evol. 2018 Mar 22;50:9. doi: 10.1186/s12711-018-0378-x (PMC5865298; doi:10.1186/s12711-018-0378-x)
Supplement: Supplementary file 1 — Additional file 1. Details of the in silico simulation study that was carried out to determine the appropriate window size to be used for GWSS analysis. [file 12711_2018_378_MOESM1_ESM.docx]

Additional File 1. Details of the *in silico* simulation study performed to determine the appropriate window size to be used for GWSS analysis.

The sequenced genomes of 20 European ewes were downloaded from NCBI database, 10 individuals corresponding to 4 meat sheep breeds and 10 individuals from 6 dairy breeds (Table 1). All data belong to the ISGC (International Sheep Genomic Consortium).

Table 1. Sequence data used for the *in silico* simulation study

| **Breed** | **Nº Individuals** | **Production** | **NCBI Accession number** |
| --- | --- | --- | --- |
| **Ojalada** | 2 | Dairy | SRX150351  SRX150340 |
| **Churra** | 2 | Dairy | SRX150349  SRX150288 |
| **Castellana** | 2 | Meat | SRX150344  SRX150323 |
| **Lacaune** | 1 | Dairy | SRX150291 |
| **Merino** | 3 | Meat | SRX150327  SRX150308  SRX150292 |
| **Swissalpine** | 4 | Meat | SRX150348  SRX150324  SRX150314  SRX150302 |
| **TurkishAwassi** | 2 | Dairy | SRX150333  SRX150312 |
| **Awassi** | 1 | Dairy | SRX150330 |
| **Swissmirror** | 1 | Meat | SRX150304 |
| **Sakiz** | 2 | Dairy | SRX150318  SRX150283 |

Sequence data were processed as described in the Materials and Methods section. Two pools were constructed (meat production pool and dairy production pool) achieving a genome coverage of 10x taking into account the number of individuals and the literature (Ferretti et al., 2013). For this simulation study two statistical parameters were estimated, pooled Tajima’s D and pooled $F_{ST}$, using the PoPoolation v1.2.2 program (Kofler et al., 2011a,b). These two parameters were calculated using four different window sizes: 50K, 100K, 150K and 200K. Then, the number of detected SNP for each window was estimated (Table 2). The distribution of the number of SNP/window for each pool and window size before and after removing those windows having less than 10 SNP was also illustrated (Figure 1).

Considering the results, the window size of 200K was chosen for our future studies because it was the one that gave us the highest proportion of windows with 10 or more SNP avoiding possible bias in the estimation of parameters used for the detection of selection signatures (Axelsson et al., 2013; Rubin et al., 2012).

Table 2:

| Meat Pool | No. windows | No. windows > 10 SNP | Deleted windows | Remaining windows (%) |
| --- | --- | --- | --- | --- |
| 50K | 49054 | 48848 | 206 | 0.9958 |
| 100K | 24534 | 24517 | 17 | 0.9993 |
| 150K | 16362 | 16356 | 6 | 0.9996 |
| 200K | 12276 | 12272 | 4 | 0.9997 |
| Dairy Pool | No. windows | No. windows > 10 SNP | Deleted windows | Remaining windows (%) |
| 50K | 49054 | 48886 | 168 | 0.9965 |
| 100K | 24534 | 24522 | 12 | 0.9995 |
| 150K | 16362 | 16355 | 7 | 0.9996 |
| 200K | 12276 | 12272 | 4 | 0.9997 |

Figure 1: distribution of the number of SNP/window for each pool and window size before and after removing those windows having less than 10 SNP


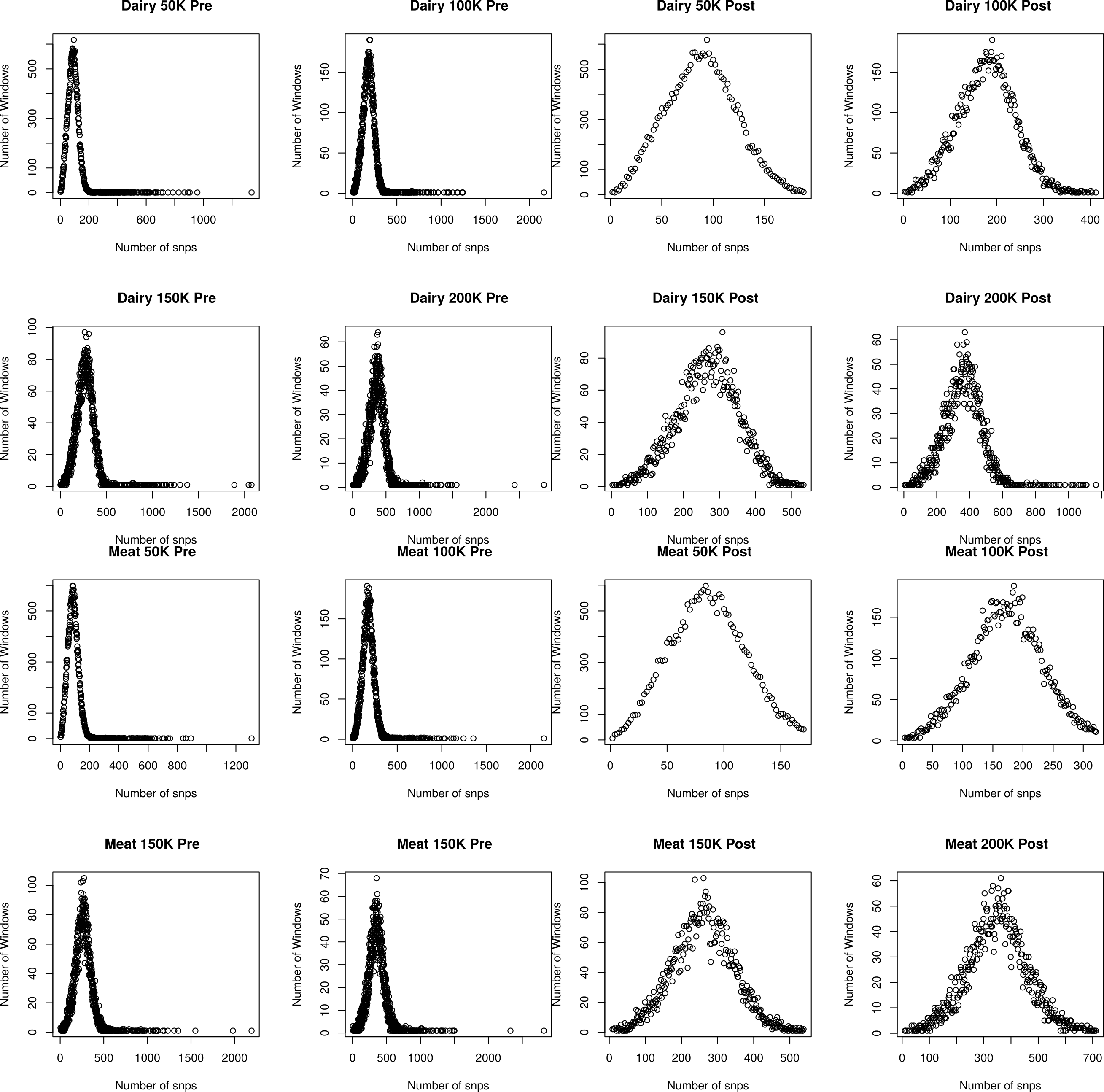


References:

Axelsson E, Ratnakumar A, Arendt ML, Maqbool K, Webster MT, Perloski M, Liberg O, Arnemo JM, Hedhammar A, Lindblad-Toh K. The Genomic Signatures of dog domestication reveals adaptation to a starch-rich diet. Nature. 2013; 495(7441):360-4.

Ferreti.L, Ramos-Onsins SE, and Pérez-Enciso.M. Population genomics from pool sequencing. Molecular Ecology. 2013; 22:5564-5576.

Kofler R, Orozco TR, Wengel P, De Maio N, Pandey RV, Nolte V, et al. PoPoolation: A Toolbox for Population Genetic Analysis of Next Generation Sequencing Data from Pooled Individuals. PLoS ONE. 2011a; 6(1):e15925.

Kofler R, VinayPandey, R, and Schloetterer, C. PoPoolation2: Identifying differentiation between populations using sequencing of pooled DNA samples (Pool-Seq). Bioinformatics. 2011b; 27(24):3435–3436.

Rubin CJ, Megens HJ, Martinez Barrio A, Maqbool K, Sayyab S, Schwochow D, Wang C, Carlborg Ö, Jern P, Jørgensen CB, Archibald AL, FredholmM, Groenen MA, Andersson L. Strong signatures of Selection in the domestic pig genome. Proc Natl Acad Sci USA. 2012; 109(48):19529-36.
